# All or nothing: No half-Merge and the evolution of syntax

**DOI:** 10.1371/journal.pbio.3000539

**Published:** 2019-11-27

**Authors:** Robert C. Berwick, Noam Chomsky

**Affiliations:** 1 Massachusetts Institute of Technology, Cambridge, Massachusetts, United States of America; 2 University of Arizona, Tucson, Arizona, United States of America

## Abstract

In their Essay on the evolution of human language, Martins and Boeckx seek to refute what they call the “half-Merge fallacy”—the conclusion that the most elementary computational operation for human language syntax, binary set formation, or “Merge,” evolved in a single step. We show that their argument collapses. It is based on a serious misunderstanding of binary set formation as well as formal language theory. Furthermore, their specific evolutionary scenario counterproposal for a “two-step” evolution of Merge does not work. Although we agree with their Essay on several points, including that there must have been many steps in the evolution of human language and the importance of understanding how language and language syntax are implemented in the brain, we disagree that there is any justification, empirical or conceptual, for the decomposition of binary set formation into separate steps.

## Abstract 2

In response to an Essay by Martins and Boeckx, this Formal Comment by Berwick and Chomsky argues that a crucial step in the evolution of human language can now be pinpointed to the appearance of a single new, but simple, computational operation.

## The evolution of syntax

In their Essay [1], Martins and Boeckx (MB) take issue with our proposals about the evolution of language in our book *Why Only Us* (WOU) [2] and offer an alternative. As we will show, their critique is misguided and their alternative untenable. But first, it is useful to clearly delineate the areas of agreement and disagreement with them.

First, in [1], MB do not question our assumption that the core properties of language are based on the combinatorial operation Merge, with the standard definition of its basic operation: binary set formation [1,2]. Our disagreement has to do with evolution of this operation. More precisely, as we shall see, the disagreement has to do with steps they propose that immediately preceded the evolution of Merge.

Second, we both agree that it is important to determine how Merge is implemented in the brain. In [2] pp. 158–164, as illustrated in Fig 4.4–4.6, we advance a specific proposal about this neural “wiring,” grounded on recent explicit neurological and comparative primate findings [3–5]. MB do not challenge this proposal. We therefore put the matter of neural implementation aside here.

Third, we both agree that it is important to determine how a Merge-based system is used, that is, how it is externalized in the sensory-motor system (typically, though not necessarily, sound) and then actually used in performance, e.g., parsing or producing sentences. MB discuss the importance of this in their discussion of David Marr’s “three levels of analysis” but advance no specific proposals as to algorithms or physical implementation. In [2], we devote nearly an entire chapter to this topic, “Triangles in the Brain,” pp. 109–166, in which we discuss both these topics, including analysis of the general architecture for such an operation and the empirical evidence for various computer science–based algorithms grounding it, and how these might crucially affect language use, all with an eye towards Marr’s distinctions about the distinct levels of algorithm and implementation that [1] also stresses. (See, e.g., [2] pp. 131–132 and pp. 135–139, for discussion of the algorithms involved, e.g., a parallel version of the conventional Cocke–Kasami–Younger algorithm, p. 136 and p. 139, see [6,7], along with an explicit proposal about the use of content-addressable memory, as this sort of memory is often considered more congenial with known neuroscience [8].) Here, too, MB’s Essay [1] offers no criticism of these proposals and, in fact, does not even mention them. It advances no specific proposals of its own on these topics. We therefore also put these topics aside here.

Fourth, we agree that there need not be, as [1] notes in its abstract, a “parallelism between the formal complexity of the operation at the computational level and the number of evolutionary steps it must imply.” As MB formulate the central point of their paper [1] p. 5: “We find it problematic to rely on ‘logical necessity’ based on the formal complexity of a trait to motivate evolutionary scenarios. It is this fallacy that we draw attention to in this paper.” We too regard it as “problematic” and, indeed, a “fallacy.” The observation is correct. We never questioned this point in our book (see, e.g., [2], p. 79, p. 164). What is under discussion is not operations in general but rather a specific one, the simplest combinatorial operation, binary set formation, called Merge. Crucially, as we discuss next, MB’s own proposal adopts our account of the evolution of Merge unchanged, thus tacitly recognizing that binary set formation (Merge) cannot be decomposed and emerges in a single step. MB then add new proposals about immediate precursors to our shared account of the evolution of Merge. The justification for the added complexities that they propose about precursors to Merge is the sole point at issue.

Finally, we both agree that it would be important to discover the long evolutionary history that preceded the appearance of Merge. In this case, although both we and [1] agree that there were multiple steps that preceded the appearance of Merge, neither we nor [1] present any explicit proposals about these previous steps, so we can put this matter aside too. The sole issue, then, on which we do disagree has to do with the evolution of the operation Merge itself, including its subcases. More precisely, as we see directly, the sole issue concerns their proposal about precursors to our shared conception of the evolution of Merge.

In [2], we proposed that the elementary operation of binary set formation (Merge) appeared in a single step, yielding automatically its two subcases. The first subcase, external Merge (EM), occurs when the two items forming the binary set {x, y} must be distinct from one another, as in [1], {the}, {book} yielding {the, book}; or {bought}, {the book}, yielding {bought, {the, book}}. The second subcase is internal Merge (IM), in which one of the items forming the binary set must be a subset of the other, as in {what}, and {bought, {what}}, yielding {what, {bought, {what}}. Note that both EM and IM are more complex than Merge, plain binary set formation, because both EM and IM explicitly contain an added constraint on top of binary set formation, namely, the “must be distinct/a subset of” clauses explicitly shown previously. So, Merge is simpler than either EM or IM in a very clear computational sense.

In [1], MB propose a single explicit, different scenario: first EM appeared and then IM, each more complex than Merge, as we have just seen. But—crucially—these added complexities still do not yield Merge. That requires a further step in the evolutionary process, yielding Merge—of course, in a single step—incorporating without comment the two more complex subcases that had allegedly emerged earlier. Note, crucially, that their proposal implicitly assumes that Merge appeared in exactly the way we describe: in a single step, following the appearance of the two precursors they postulate. One might suggest that IM appeared first, then EM, and there are even more complex proposals as to immediate predecessors of the simplest operation, Merge, though we again stress that MB advance only one explicit multistep proposal. If they have other multistep proposals in mind, they do not present them in [1]. But evidently, reasons would be needed to entertain a proposal about the evolution of Merge that is more complex than the simplest one, namely, the one we put forth. The proposal in [1], furthermore, is thus a new proposal that in fact incorporates our proposal, unchanged. To put it simply, we are now considering a theory T′ (the one in [1]), which incorporates unchanged all the assumptions of a theory T (the one we proposed in [2]), differing from T only in that it adds a number of new complex assumptions. Plainly, reasons—in fact, strong reasons—are required to entertain T′ as a possibility.

MB offer possible reasons in their Essay, but they are based on misunderstandings of formal languages and the operation Merge. The proposal in [1] rests entirely on properties of the "Chomsky hierarchy" of rewriting rules that was developed in the 1950s ([1] p. 5, Table 1, and p. 6 labeled as “The Hierarchy of Formal Languages”; see, e.g., [9]). The hierarchy adapts to language the logician E. L. Post’s general theory of computability, based on “rewriting systems”: rules that replace linear strings of symbols with new linear strings. For instance, one such rewriting rule is “VerbPhrase → Verb Determiner Noun” stating that the symbol “VerbPhrase” can be written as the linear string concatenation of three symbols, “Verb,” “Determiner,” and “Noun” in that order, ultimately—as in, e.g., “bought the book.” (Specifically, such rule systems constitute the Type 2 systems in Table 1 of [1].) As one can see, by definition, all of the “formal languages” generated at the various levels of this hierarchy involve linear order [9]. In contrast, again, by definition, Merge-based systems have (hierarchical) structure but no linear order, an essential property of binary sets formed by Merge. MB themselves illustrate this with their own example, {bought, {the, book}} ([1], p. 1), [10], a hierarchical structure with no linear order. Accordingly, Merge-based systems do not even appear in the hierarchy, and anything concluded from the study of the Chomsky hierarchy is totally irrelevant to the evolution of Merge-based systems. That is the basic reason why the sole evolutionary proposal in [1] is not tenable. This is quite apart from the fact this proposal is superfluous, as we noted previously, because it simply amounts to an added complication about precursors to Merge that have no empirical justification.

We should add that there are strong empirical reasons why the core combinatorial operation for language should keep to hierarchical structures as in the aforementioned example, {bought, {the, book}}, lacking linear order. In [2], we discuss the empirical evidence for this distinction, some of it tracing back to the 1970s, when the issues began to become clear (for one example, see [2], pp. 117–118, and Fig 4.2; as for biological evidence supporting this position, e.g., the limitations to restricted kinds of linear strings in birdsong but not human language, see [11,12]). The Essay in [1] ignores this topic completely. In fact, as noted previously, the Essay does not contest that the core properties of language are based on Merge, an operation that crucially provides only binary set hierarchically structured expressions without any linear order, radically different in principle from the systems [1] discusses in connection with “The Hierarchy of Formal Languages”.

That is enough to point out that the sole proposal in [1] about the evolution of language and its critique are mistaken. There are, however, some further errors in [1] that may be useful to disentangle.

Consider MB’s argument that EM might have emerged first, then IM, and finally, the simpler operation Merge that automatically incorporates both as subcases. The argument is based on the claim that EM accounts for nested dependencies (at a low level of the “Hierarchy of Formal Languages” Table 1, line 2), whereas IM emerged later to account for crossing dependencies (at a higher level of the hierarchy, [1] Table 1, line 3); see Fig 1 in [1]. Let us take this proposal apart step by step.

First, MB’s empirical claim linking the difference between EM and IM to “nested” and “overlapping” dependency types (see [1], Fig 1) is false. As is familiar from current introductory textbooks in linguistics (e.g., [13,14]), IM enters into generation of even the simplest nested dependencies that occur in Merge-based theories—for example, in the sentence, “where are you going,” as shown in [13], pp. 324, Fig 23, or [14] p. 357, which displays a hierarchical form with nested dependencies constructed purely by IM that we can write out in the equivalent set notation that MB [1] p. 1 adopt as {where_2_, {are_1_, {you, {are_1_, {going, where_2_}}}}}. (Here, we use the numeric subscripts on “where” and “are” purely for readability, to indicate the dependency links as in Fig 1 of [1].) Here, IM takes the two binary sets {are_1_, {you, {are_1_, {going, where_2_}}}} and {where_2_}, with the second set, {where_2_} clearly a subset of the first, {are_1_, {you, {are_1_, {going, where_2_}}}}}, and forms a result with nested dependencies, as indicated by the numeric subscripts, {where_2_, {are_1_, {you, {are_1_, {going, where_2_}}}}}.

In short, IM produces nested dependencies quite commonly, in the simplest, everyday sorts of sentences. In fact, according to widely adopted analyses appearing in standard introductory linguistics textbooks such as [15], IM operates even in simple subject–predicate sentences like “Several men are sick” ([15] example 92). According to the actual linguistic evidence, then, it doesn’t make sense to say that either EM or IM evolved first. Rather, just as we might expect if Merge evolved as a unitary operation, even simple sentences—on textbook accounts, most sentences—contain mixtures of both IM and EM deriving nested dependencies.

Second, MB claim that EM is computationally simpler than IM and therefore might reasonably be assumed to have evolved first. But this computational claim is also mistaken. Perhaps [1] had in mind some notion of parsing or recognition complexity, which again relates back to the Chomsky hierarchy and to the use of language rather than to the evolution of Merge, the topic under discussion. However, in terms of generative complexity, EM is more complex than IM. EM requires massive search to locate the elements to be merged. It requires search through the entire dictionary of the language, as well as through the set of syntactic objects already constructed, which can grow without limit as sentences become more complex. In contrast, IM requires search of only a single syntactic object. This is easily seen in the example we gave previously for “where are you going.” To apply IM to this example with {are_1_, {you, {are_1_, {going, where_2_}}}}, one needs to search at most only the expression {are_1_, {you, {are_1_, {going, where_2_}}}} to locate {where} as a subset internal to the entire expression and so a possible input to IM. This is simpler than EM because it involves searching a single, shorter, preexisting list, in a bounded, deterministic way.

In sum, there seems to be no support for the position that EM emerged before IM. The underlying reason for this confusion may possibly trace back to a misunderstanding of the relevance of the “Chomsky hierarchy” for Merge-based systems. The Chomsky hierarchy is based on concatenations of strings, with inherent linear order [9]. However, Merge-based systems are grounded on binary order-free sets, not string concatenation [10,11]. One cannot conflate the two. Therefore, any conclusions that [1] draw from this false conflation are also flawed.

The errors in [1] concerning emergence of EM and IM are, however, secondary. The crucial point is that the sole proposal in [1] about evolution of language is untenable. The “no half-merge fallacy” analysis in [1] collapses because there is no such fallacy.

## Abbreviations

EM: external merge
IM: internal merge
MB: Martins and Boeckx
WOU: *Why Only Us*
